# Superior accuracy in knee double level osteotomy using a novel hybrid fixation technique compared to conventional double plating

**DOI:** 10.1002/jeo2.12081

**Published:** 2024-07-15

**Authors:** Ahmed Mabrouk, Maureen Monda, Lucy Bell, James Broderick, Matthew Dawson

**Affiliations:** ^1^ Trauma & Orthopaedics Department Leeds Teaching Hospitals Leeds UK; ^2^ Trauma & Orthopaedics Department North Cumbria NHS Trust North Cumbria UK

**Keywords:** distal femoral osteotomy, double‐level knee osteotomy, high tibial osteotomy, hybrid fixation, intramedullary nail

## Abstract

**Purpose:**

This study aimed to compare two different double‐level knee osteotomy (DLO) fixation techniques. The primary outcome reported the radiological coronal plane correction and its accuracy. The secondary outcomes reported the correction outliers, the clinical outcomes, the 5‐year postoperative satisfaction and the complications.

**Methods:**

A retrospective review of a single surgeon osteotomy database identified 52 cases of DLO between 2011 and 2019, of which 24 cases met the inclusion criteria. Patients were categorised into two groups: the nail‐plate (NP) group fixed with a magnetic extendable intramedullary tibial nail and femoral conventional plate, and the double‐plate (DP) group fixed with conventional plates (tibia and femur). Radiographic parameters were recorded, including the mechanical femorotibial angle (mFTA), medial proximal tibial angle (MPTA), mechanical lateral distal femoral angle (mLDFA), joint line convergence angle (JLCA) and weight‐bearing line ratio (Mikulicz %). Surgical accuracy was calculated as the difference between the achieved and the planned correction. Outliers were defined as those with a greater than 10% difference from the planned correction. Simple knee value scores and visual analogue scale for pain were recorded preoperatively and postoperatively at 2 and 5 years. Five‐year patient satisfaction was recorded.

**Results:**

A total of 24 patients were included: the NP group (*n* = 12) and the DP group (*n* = 12). Significant coronal plane corrections were achieved in the NP group for the mean mFTA (preoperative 167.9° ± 3.4° to postoperative 182.1° ± 1.4°), the mean MPTA (preoperative 83.5° ± 2.9° to postoperative 91.3° ± 2.8°) and the mean mLDFA (preoperative 89.8° ± 3.4° to postoperative 85.9° ± 4.4°). Similarly, significant coronal plane corrections were achieved in the DP group for the mean mFTA (preoperative 168.6° ± 4.4° to postoperative 182.2° ± 2°), the mean MPTA (preoperative 84.2° ± 2° to postoperative 88.3° ± 4.1°) and the mean mLDFA (preoperative 90.7° ± 2.9° to postoperative 83.9° ± 1.7°) (all *p* < 0.05). The mean correction accuracy was higher for the NP versus DP group at 3.4 ± 3.4% versus 7.1 ± 3.9% (intergroup *p* < 0.05). There were no outliers in the NP group versus two outliers (overcorrected) (16.7%) in the DP group. Significant clinical improvement was reported in both groups at 2 and 5 years postoperatively (all *p* < 0.05).

**Conclusion:**

Superior correction accuracy and no outliers were achieved in hybrid fixation double‐level knee osteotomy compared to the conventional double‐plating technique. The magnetic extendable nail offers the advantage of fine‐tuning the correction postoperatively and could be a potential research template for future designs of postoperative correction implants.

**Level of Evidence:**

Level III, retrospective cohort study.

**Abbreviations**
DFO: distal femoral osteotomy
HTO: high tibial osteotomy
JLCA: joint line convergence angle
LSRs: long leg standing radiographs
mFTA: mechanical femoro‐tibial angle
mLDFA: mechanical lateral distal femoral angle
MOW: medial opening wedge
MOWHTO: medial opening wedge high tibial osteotomy
MPTA: medial proximal tibial angle
OA: osteoarthritis
SKV: simple knee value
VAS: visual analogue scale

## INTRODUCTION

Knee preservation surgery has witnessed a rapid evolution of osteotomy techniques over the past two decades [4, 9, 21, 36, 48, 56]. Medial unicompartmental knee osteoarthritis (OA) may be managed with a high tibial osteotomy (HTO) [30, 31], distal femoral osteotomy (DFO) [17, 39] or double‐level osteotomy (DLO) [4].

The success of knee osteotomy relies on the correction accuracy which translates into improved clinical outcomes [20, 22]. While there is no current consensus on an ideal correction target [13], an optimal postoperative lower limb alignment can be judged by the mechanical tibiofemoral angle and the weight‐bearing line ratio (Mikulicz %) [8, 41].

Several adjuncts have been introduced throughout the osteotomy process to improve accuracy, such as digital planning software, three‐dimensional planning, patient‐specific instrumentations and computer‐assisted navigation [5, 13, 45]. Nevertheless, post‐osteotomy correction errors are still being repeatedly reported [35, 49], even with computer‐assisted navigation [52] and with accurately achieved intraoperative alignment [26].

HTO using a magnetic extendable intramedullary tibial nail (ME‐IMN) has demonstrated a proof of concept with results similar to those achieved with a fixed angle plate system [23]. However, in cases of substantial or bifocal deformities, a single‐level osteotomy may significantly increase the joint line obliquity (JLO) with resultant tibiofemoral subluxation, pressure distribution changes and excessive shear stresses on the articular cartilage [2, 34, 37, 54]. In such cases, DLO is gaining popularity over single‐level knee osteotomy due to its merits in maintaining a more physiologic joint alignment and orientation with better clinical results [1, 7, 47] and comparable return to sports [6, 12]. Nevertheless, DLO is a complex procedure, and accuracy is of the essence.

This study compares hybrid fixation DLO (tibia ME‐IMN and femur conventional plate) to the conventional double plating technique of the tibia and the femur. The primary outcome reported the radiological coronal plane corrections and their accuracy. The secondary outcomes reported the correction outliers, the clinical outcomes, the 5‐year postoperative satisfaction and the complications. It was hypothesised that the novel hybrid fixation technique would yield equivalent results in accuracy and clinical outcomes compared to the conventional double plate fixation.

## METHODS

**Figure 1:**
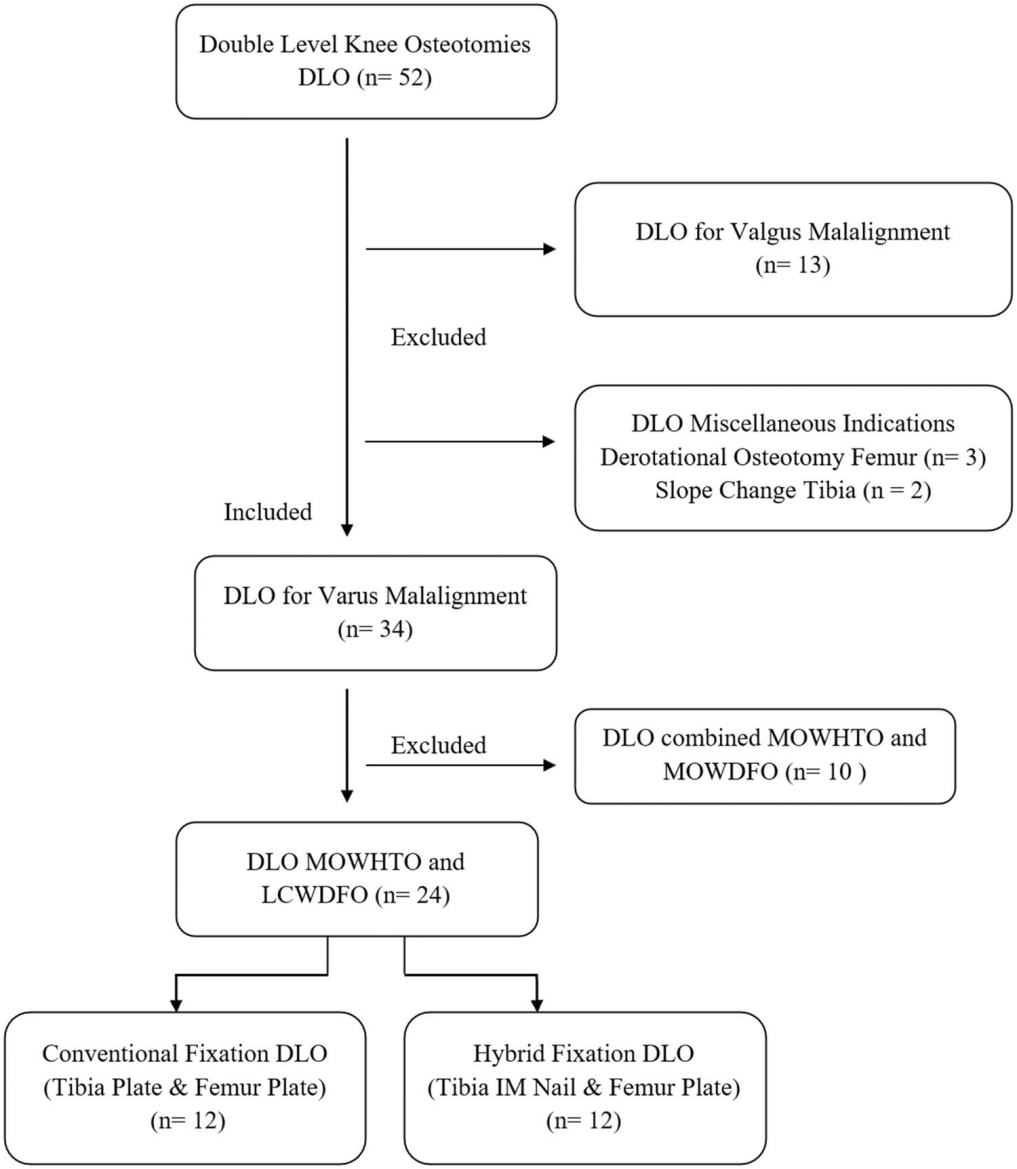
Patient flow chart. DLO, double level osteotomy; LCWDFO, lateral closing wedge distal femoral osteotomy; MOWDFO, medial opening wedge distal femoral osteotomy; MOWHTO, medial opening wedge high tibial osteotomy.

A retrospective review of a prospective single‐surgeon database was undertaken. All patients consented to inclusion in the database and future research. Fifty‐two consecutive DLO cases were identified between 2011 and 2019, of which 24 were included in the study. Inclusion criteria were patients ≥18 years old with no maximum age, symptomatic medial knee OA (Kellgren Lawrence I–IV) that failed conservative management, varus knee malalignment and indications for double‐level knee osteotomy. Exclusion criteria were valgus knee malalignment, single‐level knee osteotomy, either tibial or femoral, varization osteotomies, and derotational, multiplanar and sagittal plane corrections. The nail manufacturer (Nuvasive Specialised Orthopaedics) advised an upper weight limit of 115 kg, which was observed without the need for exclusions in the presented series. DLO was indicated for cases where preoperative planning with isolated HTO resulted in postoperative MPTA of ≥95° or cases with larger corrections with the existence of significant bifocal deformities (tibia and femur). Patients were categorised into two groups; the nail‐plate (NP) group, where fixation was performed with tibia ME‐IMN and femur plate, and the double‐plate (DP) group, where fixation was performed with tibia and femur plate. Figure 1. There were no clinical indications for allocating patients to either fixation technique. All patients in the National Health Service (NHS) sector were treated with hybrid fixation after proof of concept was established for the use of the device in single‐level osteotomy (HTO) until the conclusion of the study period, while the double plate technique was used for all patients in the private sector. The Nuvasive nail had been successfully used in the NHS in a previous study [23]. Whereas, in the private hospital sector, the insurance companies would not underwrite the extra cost of the magnetic nail.

### Clinical and radiographic assessment and surgical planning

Preoperatively, all patients had anteroposterior long leg standing radiographs (LSRs) and deformity analysis was performed with the calculation of the mechanical Femoro‐Tibial Angle (mFTA), the medial proximal tibial angle (MPTA), mechanical lateral distal femoral angle (mLDFA), joint line convergence angle (JLCA) and weight‐bearing line ratio (Mikulicz %). The same measurements were calculated 3 months postoperatively. All measurements were performed by the senior author (Matthew Dawson) according to Paley's principles [40]. Planning was performed by using Miniaci's method for the MOWHTO and the reversed Miniaci method for the LCWDFO. Both planning methods have been reported to have good to excellent inter‐ and intra‐rater reliability [16, 17].

Patients were clinically assessed by the simple knee value (SKV) scores [32] and the visual analogue scale (VAS) for pain preoperatively and 2 and 5 years postoperatively. Patient satisfaction was recorded at 5 years postoperatively.

**Figure 2:**
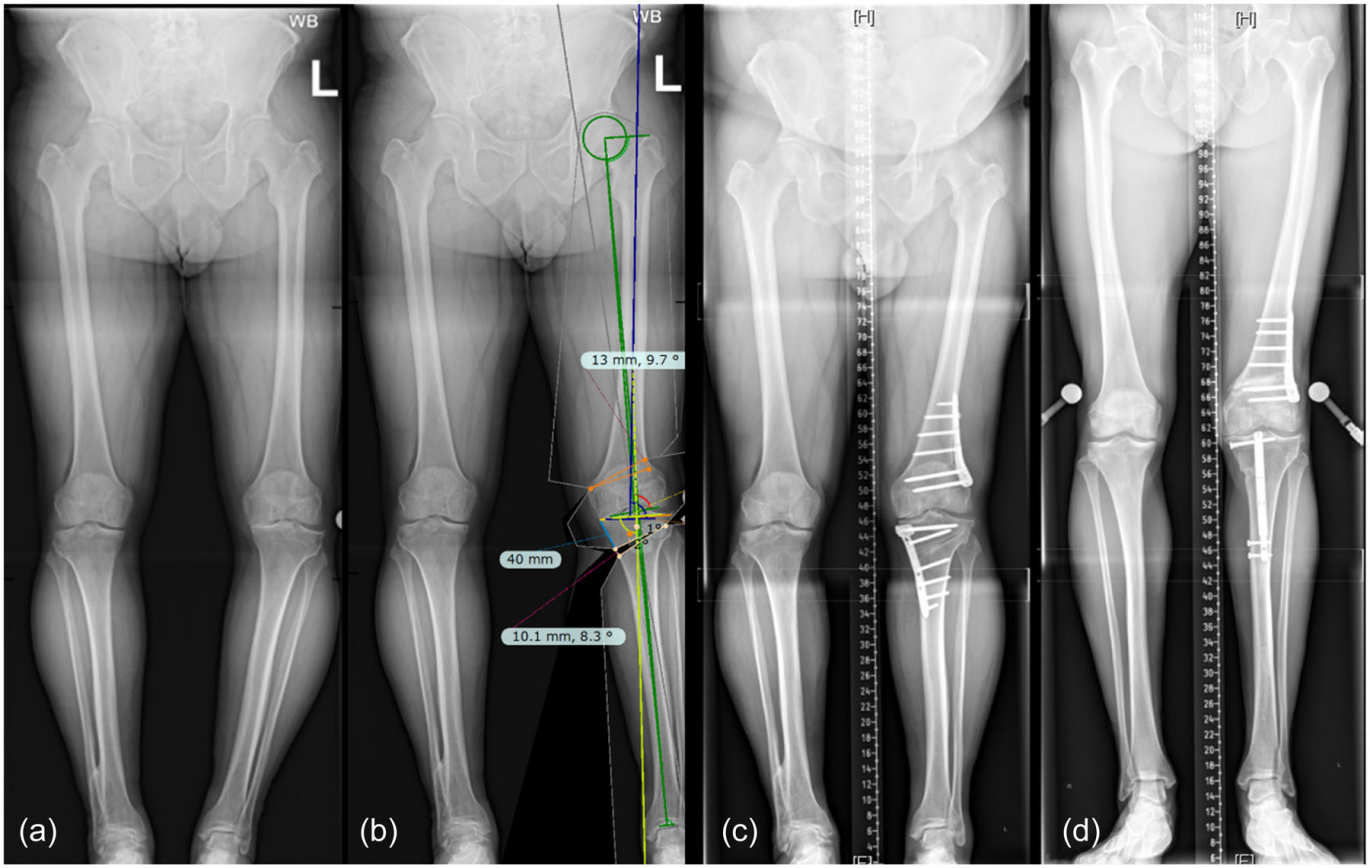
Anteroposterior bilateral long leg standing radiographs. (a) Showing bilateral knee osteoarthritis with varus malalignment (more severe on the left side). (b) Preoperative trauma‐cad simulation of the alignment following DLO on the left side. (c) Postoperative left DLO (MOWHTO and LCWDFO) demonstrating double plate fixation of the tibia and the femur. (d) Postoperative left DLO (MOWHTO and LCWDFO) demonstrating tibial fixation with IM extendable magnetic nail and femoral fixation with a plate. DLO, double level osteotomy; LCWDFO, lateral closing wedge distal femoral osteotomy; MOWDFO, medial opening wedge distal femoral osteotomy; MOWHTO, medial opening wedge high tibial osteotomy.

Osteotomy planning was undertaken with digital planning software TraumaCad® (BrainLAB AG) using a modified Miniaci method [16, 24] with the planned Mikulicz set at 55% from the medial tibial plateau. Figure 2. The DLO was planned so that the postoperative values for both the mLDFA and MPTA would fall between 85° and 90° for both parameters. If one or both values were to fall outside of this range, then every effort was made to prioritise the MPTA so that this value would not exceed 94°.

### Surgical procedure

A standard surgical protocol was followed for both DFO and HTO [11, 29, 55]. All cases had a combination of a lateral closing wedge distal femoral osteotomy (LCWDFO) and a medial opening wedge high tibial osteotomy (MOWHTO). Biplanar osteotomies were performed in all the cases [10, 42]. A fixed‐angle plate system was used for all DFO fixation and HTO fixation in the DP group.

**Figure 3:**
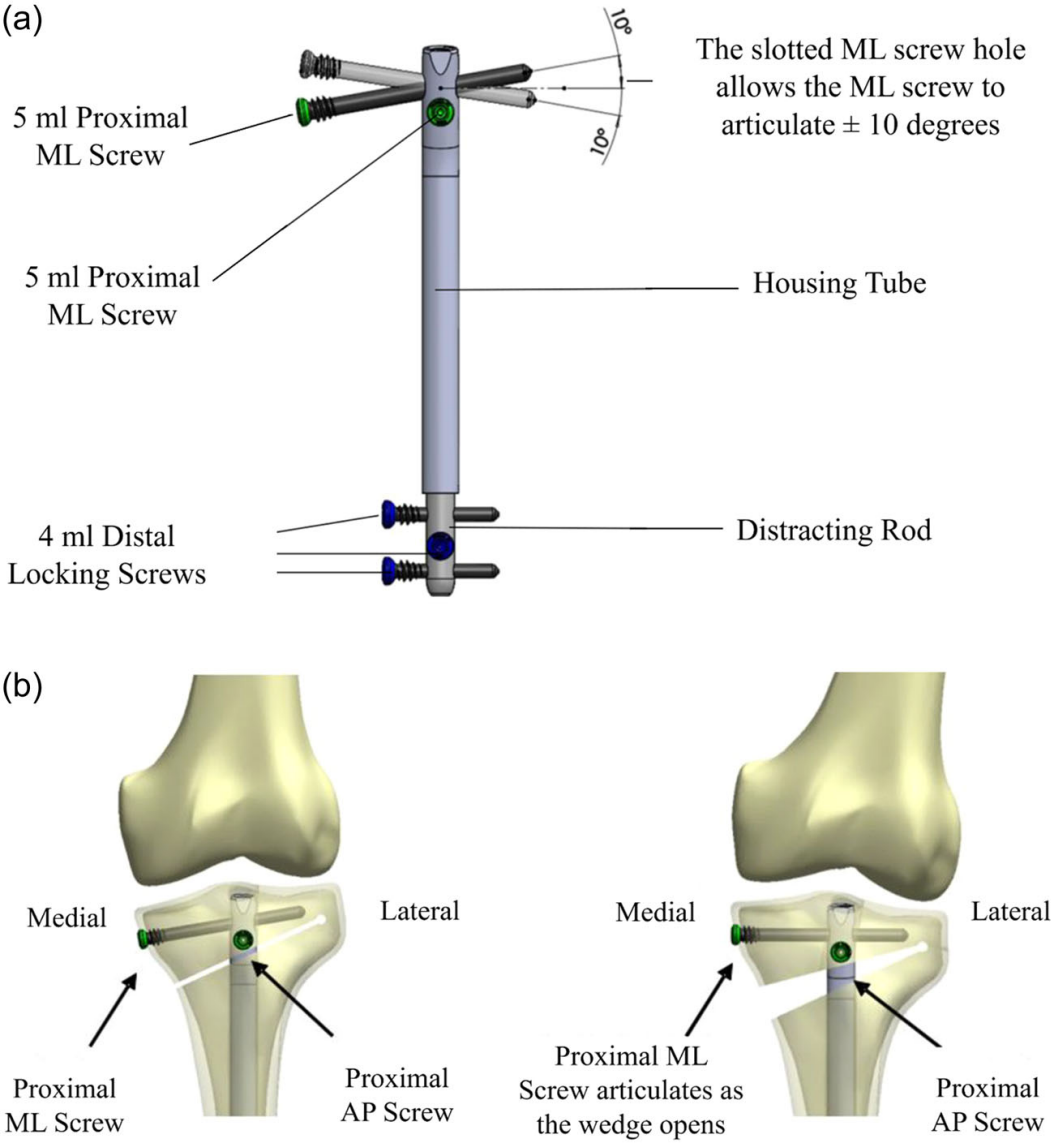
Graphic representation of the OPTY‐LINE® nail (Nuvasive Specialised Orthopaedics) for high tibial osteotomy, demonstrating (a) The locations of the four screws for fixation and the housing tube containing a magnet, gears and threaded pin, which is distracted in stages postoperatively. Image courtesy of Nuvasive Specialised Orthopaedics. (b) The proximal tibia and the knee joint in the immediate postoperative period (left) and 6 weeks postoperatively (right) following rod distraction within the OPTY‐LINE device. Image courtesy of (Nuvasive Specialised Orthopaedics). AP, anterio‐posterior; ML, medial–lateral.

In the NP group, the intramedullary device was the Nuvasive PRECICE OPTY‐LINE® nail (Nuvasive Specialised Orthopaedics). A longitudinal paramedian incision elongated distally and allowed access for the proximal tibia to perform the HTO. Initially, the nail track was established, a trial nail was inserted, and the position of the proximal cortical anteroposterior screw was predrilled [23]. The trial nail was then removed, and a bi‐planar osteotomy was performed [11, 29]. A dynamic rehearsal of the opening wedge osteotomy was performed by slowly and incrementally opening the gap to 50% over the estimated target requirement. This was mainly to ensure that the ultimate distraction in the postoperative phase was well within the rehearsed tolerance of the construct to avoid potentially excessive harmful stresses on the hinge or the screws during the distraction process. Once the MOWHTO was performed, the actual nail was inserted, and proximal and distal locking was performed with two screws each, as shown in Figure 3.

Appropriate planning of the nail lengthening process required the calculation of approximately two‐thirds of the planned opening gap at the medial cortex as a representative amount of the distraction to be performed by the nail to achieve the same degree of opening. A short latent period of up to 7 days preceded the commencement of nail distraction. The typical regime for the nail distraction would last approximately 2 weeks and was individually tailored to each case. This involved 0.5 mm lengthening per day and, therefore, 7 mm over 14 days. The resultant opening at the medial cortex would, therefore, approximate 10 mm in this scenario. However, depending on the individual prescription, the duration of distraction might be longer or shorter than 14 days. At 3 weeks postoperation, LSRs were performed to assess the osteotomy, the new mechanical axis, and the new Mikulicz point. If under or over‐correction was witnessed at this stage, an appropriate schedule was introduced for the coming days involving either lengthening or shortening the device in small increments. A further radiographic assessment was performed at 5–6 weeks postoperatively. In all cases, the targeted Mikulicz point was set at 55% with a tolerance of 10% in either direction. Wherever possible and especially early in the process, a small overcorrection was preferred to an under‐correction in anticipation of a tendency for a small degree of bony compression to occur during the healing process in the absence of rigid fixation. Patients were trained to use the machine and were supervised at home remotely by a senior member of the Idealmed Company.

### The mechanism of correction with the extendible IM magnetic nail

The ME‐IMN technique is designed for the use in MOWHTO in varus knees. In the cases where the tibial osteotomy was fixed with the ME‐ IMN, opening and closing of the osteotomy gap was controlled by lengthening and shortening of the device in the intramedullary cavity. An external remote control is rested on the leg by the patient. This remote device contains magnets that produce a magnetic field which in turn causes a magnetised rare earth metal (neodymium) to rotate within the nail. As this rotation occurs, an intricate internal gearing system converts the rotational movement into either distraction or compression of the nail, thus opening and closing the osteotomy gap until the preplanned Mikulicz correction point is achieved, as shown in Figure 3. The external remote control is simply turned through 180° by the user to reverse the direction of rotation and, therefore, lengthen or shorten. The nail and its control device share technology used in the PRECICE nail [25]. In the experience of the senior author, corrective movements of the nail have been performed successfully for up to 90 days postsurgery and have been reported in Kirane et al. [25].

### Postoperative management

Postoperatively, all patients followed a standard protocol of toe‐touch weight bearing with crutches for two weeks, allowing for a full range of knee motion (closed chain exercises). Thereafter, they progressed to partial weight‐bearing and were instructed to fully weight‐bear at 6 weeks.

In addition to the 2‐weekly visit for the nail patients, all patients were reviewed at approximately 6 and 12 weeks postsurgery and evaluated with LSRs at the later visit. Patients in the NP group had further surveillance radiographs as required. Patients in the NP group had an average of 5.3 radiograph visits compared to 2.5 visits of patients in the DP group. TraumaCad® was used to assess the accuracy of correction and changes in measurements of preoperative parameters.

### Surgical accuracy and outliers

The correction accuracy was calculated as the absolute deviation of the achieved correction from the planned correction [44]. Outliers were those cases with greater than 10% difference from the planned correction.

### Statistical analysis

Data were analysed by using statistical software (R Core Team, 2022, R Foundation for Statistical Computing). Descriptive statistics were reported as means ± standard deviations (95% confidence intervals). The Shapiro–Wilk test was used to check the normality of data distribution. Student's paired *t*‐tests and Mann–Whitney *U* tests were used for intergroup and intragroup comparisons. Statistical significance was assumed at *p*‐values of <0.05.

## RESULTS

A total of 24 patients were included: the NP group (*n* = 12) and the DP group (*n* = 12). The mean age was 53.8 ± 7.6 years in the NP group versus 53.9 ± 8.2 years in the DP group (n.s). The mean BMI was 26.3 ± 4.2 kg/m^2^ in the NP group versus 31.3 ± 5.7 kg/m^2^ in the DP group (n.s) (Table 1).

**Table 1:** Patient demographics.

Variable	Nail‐plate group (*n* = 12)	Double‐plate group (*n* =12)	Intergroup (*p*‐value)
Age (years)	54.9 ± 6.9	53.8 ± 8.6	0.9
	(51, 58.8)	(48.7, 58.9)	
BMI (Kg/m^2^)	25.7 ± 3.4	31.6 ± 6.1	0.2
	(23.7, 28.9)	(27.4, 35.3)	
Gender			
Male	9	11	
Female	3	1	
Side			
Left	5	7	
Right	7	5	

*Note*: 95% confidence interval in brackets.

Abbreviations: BMI, body mass index; Kg/m2, kilogram per metre squared.

Coronal plane corrections were reflected by significant changes in the mFTA, MPTA and mLDFA in the NP group from preoperative means of 167.9° ± 3.4°, 83.5° ± 2.9° and 89.8° ± 3.4° to postoperative means of 182.1° ± 1.4°, 91.3° ± 2.8° and 85.9° ± 4.4°, respectively. Corrections in the DP group were from preoperative means of 168.6° ± 4.4°, 84.2° ± 2° and 90.7° ± 2.9° to postoperative means of 182.2 ± 2°, 88.3° ± 4.1° and 83.9° ± 1.7°, respectively. (All *p* < 0.001) Table 2. There were no intergroup significant differences between the mean preoperative and postoperative values of all alignment parameters (n.s).

**Table 2:** The mean preoperative and postoperative radiographic measurements, along with the absolute delta values and *p*‐values for all patients.

	Nail‐plate group				Double‐plate group			
Parameter	Preoperative	Postoperative	Delta value	*p*‐Value	Preoperative	Postoperative	Delta value	*p*‐Value
mFTA (degree)	167.9 ± 3.4	182.1 ± 1.4	14.3 ± 3	<0.001	168.6 ± 4.4	182.2 ± 2.0	13.6 ± 3.6	<0.001
	(166, 169.8)	(181.3, 182.9)	(12.6, 16)		(166.5, 171.2)	(181.0, 183.4)	(11.5, 15.7)	
mLDFA (degree)	89.8 ± 3.4	85.9 ± 4.4	−3.9 ± 4.6	0.01	90.7 ± 2.9	83.9 ± 1.7	−6.8 ± 3.1	<0.001
	(87.9, 91.8)	(83.4, 88.4)	(−6.5, −1.3)		(89, 92.4)	(82.9, 84.9)	(−8.7, −5.0)	
MPTA (degree)	83.5 ± 2.9	91.3 ± 2.8	7.8 ± 3.6	<0.001	84.2 ± 2	88.3 ± 4.1	4.1 ± 3.6	0.002
	(81.9, 85.1)	(89.8, 92.9)	(5.8, 9.9)		(83, 85.4)	(85.8, 90.7)	(1.9, 6.2)	
JLCA (degree)	5.7 ± 3.3	4.5 ± 2.7	−1.2 ± 1.5	0.01	4.7 ± 2.1	3.6 ± 1.6	−1.1 ± 2.0	0.09
	(3.8, 7.5)	(2.9, 6.1)	(−2.0, −0.3)		(3.5, 6.0)	(2.7, 4.6)	(0.08, 2)	
MAD (mm)	46.7 ± 12.1	6.8 ± 4.4	39 ± 14.3	<0.001	43.5 ± 14.7	7.1 ± 5.8	−36.5 ± 18	<0.001
	(39.8, 53.5)	(4.4, 9.3)	(−48.3, −31.4)		(34.9, 52.2)	(3.7, 10.5)	(−47.1, −25.8)	
Mikulicz (%)	−5.6 ± 15.4	58 ± 3.8	63.7 ± 13.9	<0.001	0.7 ± 17.1	58.1 ± 7.8	57.3 ± 14.2	<0.001
	(−14.3, 3.1)	(55.9, 60.2)	(55.8, 71.5)		(−9.4, 10.8)	(53.5, 62.7)	(49, 65.7)	

*Note*: 95% confidence interval in brackets.

Abbreviations: JLCA, joint line convergence angle; MAD, mechanical axis deviation; mFTA, mechanical Femorotibial angle; mLDFA, mechanical lateral distal femoral angle; MPTA, medial proximal tibial angle.

### Surgical accuracy and outliers

**Figure 4:**
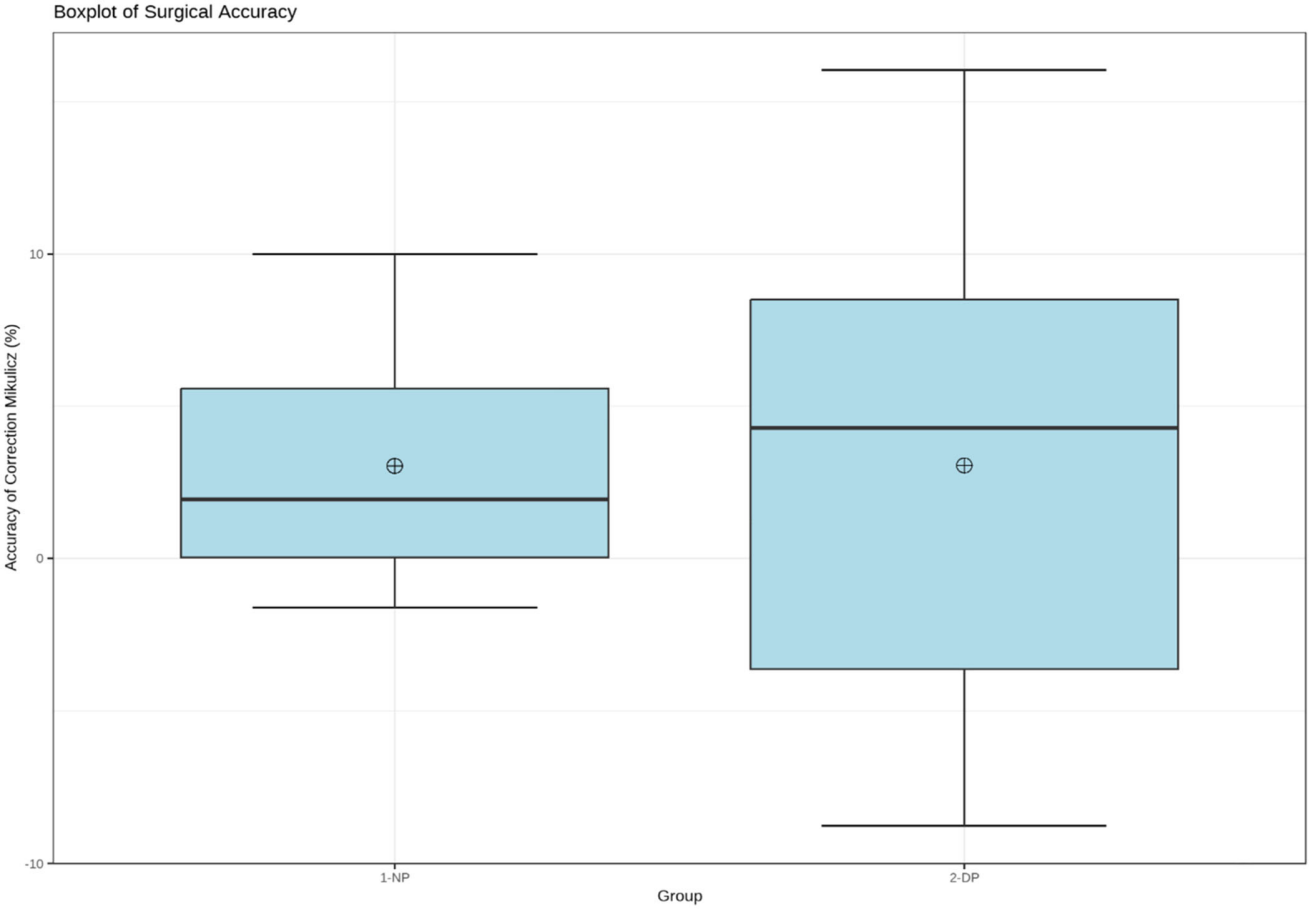
Side‐by‐side boxplots for the NP (nail‐plate) group and the DP (double‐plate) group demonstrate the significantly superior accuracy of the NP group compared to the DP group. The mean (circle with +) and median (middle line) are represented.

The mean correction accuracy of the NP group at 3.4 ± 3.4% (95% CI: 1.5, 5.4) was higher than the mean correction accuracy of the DP group at 7.1 ± 3.9% (95% CI: 4.9, 9.4), with significant intergroup difference (*p* < 0.05). There were no outliers in the NP group versus two outliers (overcorrected) (16.7%) in the DP group (Figure 4).

Clinically, all patients in both groups were improved as reflected by significant improvement in the SKV and VAS pain scores at 2 and 5 years postoperatively. There were no statistically significant in‐between group differences for the delta values of either the SKV scores at 2 years (n.s) and 5 years (n.s), and the VAS pain scores at 2 years (n.s) and 5 years (n.s) Table 3. The 5‐year postoperative satisfaction in the NP group was 83.4 ± 28, and in the DP group was 86.9 ± 25.6, with no statistically significant difference (n.s). There was an overall 12.5% complication rate, which included two cases in the NP group and one case in the DP group. Table 4.

**Table 3:** The mean preoperative and postoperative values (2 and 5 years) of the simple knee value and VAS pain scores, along with the absolute delta values and *p* values.

	Preoperative	Postoperative (2 years)	Delta value (2 years)	*p*‐Value	Postoperative (5 years)	Delta value (5 years)	*p*‐Value
Simple knee value scores							
Nail‐plate group	24.3 ± 17.2	81.4 ± 15.7	57.1 ± 17	0.002	66.4 ± 33.5	42.1 ± 25.5	0.02
	(11.6, 37)	(69.8, 93.1)	(44.5, 69.8)		(41.6, 91.3)	(23.3, 61)	
Double‐plate group	31.3 ± 18.1	88.1 ± 9.2	56.9 ± 17.5	<0.001	80 ± 23.5	48.8 ± 33	0.004
	(18.7, 43.8)	(81.7, 94.5)	(44.7, 69)		(63.8, 96.3)	(25.9, 71.6)	
VAS pain scores							
Nail‐plate group	83.4 ± 17.3	17.1 ± 20.6	66.3 ± 21.9	0.003	19.3 ± 20.5	64.1 ± 23.5	0.003
	(70.6, 96.3)	(1.9, 32.4)	(82.5, 50)		(4.1, 34.5)	(81.5, 46.8)	
Double‐plate group	91.3 ± 11.3	18.6 ± 25.9	72.5 ± 27.7	<0.001	23.8 ± 21.3	67.5 ± 27.1	<0.001
	(83.5, 99)	(0.8, 36.7)	(91.7, 53.3)		(9, 38.5)	(86.3, 48.7)	

*Note*: 95% confidence interval in brackets.

Abbreviation: VAS, visual analogue scale.

**Table 4:** Complications and their treatment in both groups.

Group	Complication	Management
NP	Proximal AP screw back‐out (*n* = 1) (no loss of correction)	Screw replacement at 4 weeks postoperative (to avoid compromising nail functionality)
	Proximal ML screw malposition (*n* = 1) (anterolateral tibial cortex penetration)	Screw removal at 6 months postoperative (osteotomy united)
DP	Superficial surgical site infection (*n* = 1)	Surgical debridement and antibiotics course

Abbreviations: AP, anteroposterior; DP, double‐plate group; ML, mediolateral; NP, nail‐plate group.

## DISCUSSION

The most important finding in the presented study is the demonstration of significant coronal plane correction achieved by two different DLO fixation techniques and the superior surgical correction accuracy in the hybrid fixation‐DLO technique, with tibial magnetic extendable IM nailing and conventional femoral plate, compared to the conventional double plating DLO technique.

In the presented series, the delta MFTA correction was 14.3° ± 3° in the NP group and 13.6° ± 3.6° in the DP group, which is a comparable correction to the published series [3, 46]. Schröter et al. [46] and Akaoka et al. [3] reported on a series of DLO, where the average overall coronal plane corrections were 11 and 14 degrees, respectively.

The planned Mikulicz line was projected to a point 55% from the medial tibial plateau, which corresponds to mFTA of 181° ± 2° [18]. The surgical accuracy in both groups was comparable to the published series but significantly more accurate in the NP group, 3.4 ± 3.4% versus 7.1 ± 3.9% in the DP group. These results were mainly attributed to the versatility of the nail that allows fine‐tuning the correction postoperatively.

Patients in both groups showed clinical improvement, as reflected by significant improvement in the simple knee value scores and VAS pain scores at 2 and 5 years. Additionally, the 5‐year postoperative satisfaction in the NP group was 83.4% ± 28% and in the DP group was 86.9% ± 25.6%, with no statistically significant difference. A low complication rate of 12.5% was recorded with no major surgeries required. These results are comparable to other studies published in the literature and indicate the non‐inferiority of the nail plate combination when compared to the conventional double plate technique [15].

Current practice indicates the employment of DLO to correct a large or bifocal (tibia and femur) varus deformity, where a single‐level correction might introduce significant joint line obliquity [7, 43]. However, it is a complex procedure, and one disincentive is the risk of introducing error by the addition of a further level of correction. Under‐correction can lead to failure, revision osteotomy or early conversion to arthroplasty [11, 50]. Whereas, overcorrection may lead to poor outcomes by introducing increased wear in the overloaded compartment [33, 38, 50] and may also be aesthetically displeasing to patients.

A systematic review of coronal alignment in conventional HTO demonstrated that most studies fall short of accuracy [53]. This has led to strategies that increase surgical precision intraoperatively, including the more traditional fluoroscopic confirmation [24, 57], gap measurement [24, 28], and more recently, patient‐specific instrumentation (PSI) [14, 19] and computer navigation [27].

There are many potential sources of error which may lead to impaired accuracy at each stage of osteotomy surgery. All methodologies (including gap measurement, patient‐specific instrumentation [PSI], computer navigation and fluoroscopic alignment rod evaluation) require precise preoperative planning and depend upon the variable quality of radiographs. A methodical analysis is still prone to possible inaccuracy through PACS annotation planning or the use of software platforms [18, 27, 48]. PSI [16, 22] and computer navigation [31, 48] are comparable techniques, but each still harbours potential errors. During surgery, the failure to recognise hinge fractures [51], mismeasurement of the gap or misuse of the alignment may have a significant impact. Further errors may be introduced by inappropriate postoperative rehabilitation. The ability, therefore, to reassess and alter correction angles in the postoperative phase provides an opportunity to improve accuracy and mitigate any of these sources of error.

The main limitation of the presented study is that it is neither randomised nor controlled but a series from a single high‐volume osteotomy surgeon. Additionally, the low numbers of patients make the study underpowered. The superior surgical accuracy demonstrated is based on radiographic analysis conducted at 3 months postoperatively, and therefore, correction may have subsequently been lost in some cases. This would have been enhanced by further follow‐up radiography at 2 and 5 years. On the other side, the main strength lies in the novelty of applying the technique for a complex procedure.

To our knowledge, this is the first study to report on a technique that aims to improve correction accuracy by addressing the correction in the postoperative stage for double‐level osteotomy. Future research may further investigate devices and adjuncts that might have the capability to modify or fine‐tune the correction accuracy postoperatively.

## CONCLUSION

Superior correction accuracy and no outliers were achieved in hybrid fixation double‐level knee osteotomy compared to the conventional double‐plating technique. The magnetic extendable nail offers the advantage of fine‐tuning the correction postoperatively and could be a potential research template for future designs of postoperative correction implants.

## AUTHOR CONTRIBUTIONS

All authors have participated in the content and design of the study and have seen and agreed with the contents of the manuscript.

## CONFLICTS OF INTEREST STATEMENT

Matt Dawson is an educational consultant for Bodycad. The remaining authors declare no conflict of interest.

## ETHICS STATEMENT

All patients have consented to get onto the osteotomy database and for future research purposes.

## Data Availability

All data are available upon request with approved permission from North Cumbria Hospitals.
